# Swine-Derived Probiotic *Lactobacillus plantarum* Inhibits Growth and Adhesion of Enterotoxigenic *Escherichia coli* and Mediates Host Defense

**DOI:** 10.3389/fmicb.2018.01364

**Published:** 2018-06-26

**Authors:** Jing Wang, Yanxia Zeng, Sixin Wang, Hui Liu, Dongyan Zhang, Wei Zhang, Yamin Wang, Haifeng Ji

**Affiliations:** Institute of Animal Husbandry and Veterinary Medicine, Beijing Academy of Agriculture and Forestry Sciences, Beijing, China

**Keywords:** *Lactobacillus plantarum*, ETEC, growth prevention, adhesion inhibition, host defense peptides

## Abstract

Weaning stress renders piglets susceptible to pathogen infection, which leads to post-weaning diarrhea, a severe condition characterized by heavy diarrhea and mortality in piglets. Enterotoxigenic *Escherichia coli* (ETEC) is one of typical strains associated with post-weaning diarrhea. Thus, prevention and inhibition of ETEC infection are of great concern. Probiotics possess anti-pathogenic activity and can counteract ETEC infection; however, their underlying mechanisms and modes of action have not yet been clarified. In the present study, the direct and indirect protective effects of *Lactobacillus plantarum* ZLP001 against ETEC infection were investigated by different methods. We found that bacterial culture and culture supernatant of *L. plantarum* ZLP001 prevented ETEC growth by the Oxford cup method, and ETEC growth inhibition was observed in a co-culture assay as well. This effect was suggested to be caused mainly by antimicrobial metabolites produced by *L. plantarum* ZLP001. In addition, adhesion capacity of *L. plantarum* ZLP001 to IPEC-J2 cells were observed using microscopy and counting. *L. plantarum* ZLP001 also exhibited a concentration-dependent ability to inhibit ETEC adhesion to IPEC-J2 cells, which mainly occurred via exclusion and competition mode. Furthermore, quantitative real time polymerase chain reaction (qPCR) analysis showed that *L. plantarum* ZLP001 upregulated the expression of host defense peptides (HDPs) but did not trigger an inflammatory response. In addition, *L. plantarum* ZLP001 induced HDP secretion, which enhanced the potential antimicrobial activity of IPEC-J2 cell-culture supernatant after incubation with *L. plantarum* ZLP001. Our findings demonstrate that *L. plantarum* ZLP001, an intestinal *Lactobacillus* species associated with piglet health, possesses anti-ETEC activity. *L. plantarum* ZLP001 might prevent ETEC growth, inhibit ETEC adhesion to the intestinal mucosa, and activate the innate immune response to secret antimicrobial peptides. *L. plantarum* ZLP001 is worth investigation as a potential probiotics.

## Introduction

Piglets are exposed to various stresses after weaning and are vulnerable to infections caused by enteric pathogens that cause post-weaning diarrhea. Intestinal infection severely affects piglet health and growth performance (Campbell et al., 2013) and sometimes results in mortality, thus causing considerable economic loss. Therefore, the inhibition of pathogens, especially certain strains of *Escherichia coli* (*E. coli*), responsible for significant infections in piglets, with disease forms ranging from mild to bloody diarrhea, is of special interest in the swine industry (Fairbrother et al., 2005). Enterotoxigenic *E. coli* (ETEC) is one of the main pathogens associated with post-weaning diarrhea in piglets, and ETEC infection can be fatal for piglets and leads to death in more than 50% of piglets (Gyles, 1994; Bailey, 2009).

Probiotics have been studied extensively as a main potential antibiotic alternative in animal husbandry and have been demonstrated to benefit animal health in multiple ways. Moreover, they are considered to be the only efficient feed additive against pathogen infection in piglets (Gresse et al., 2017). Probiotics have been shown to counteract ETEC-induced injury and inflammation (Guerra-Ordaz et al., 2014; Yang et al., 2015; Trevisi et al., 2017). However, not all probiotic species exert anti-infection activity in the intestines, and their underlying mechanisms are still insufficiently characterized. Their protective effect against pathogenic infections was recently shown to involve inhibition of pathogen growth, prevention of adhesion of pathogens to the intestinal mucosa, and modulation of the inflammatory responses of intestinal epithelial cells (Gresse et al., 2017).

Growth inhibition of pathogens is one of the most direct and important ways in which probiotics act against pathogens, and is considered the most essential characteristic of probiotic strains. Growth inhibition by probiotics is thought to occur mainly via a lowering of the pH to a level not suitable for most pathogens (Sreekumar and Hosono, 2000; Lin et al., 2009). Probiotics also combat pathogens by producing a variety of microbicidal substances, such as bacteriocins and microcins, which exert bactericidal or bacteriostatic actions (Dubreuil, 2017). Multiple probiotics have been demonstrated to inhibit pathogen growth by one or both of these mechanisms. Probiotics may also have the ability to reduce or prevent pathogen colonization of the animal intestine by inhibiting pathogen adhesion in a strain-specific and concentration-dependent manner (Walsham et al., 2016; Wang et al., 2016). Moreover, different probiotics employ different adhesion inhibition mechanisms, such as steric hindrance, competitive exclusion, or regulation of the immune system (Roselli et al., 2006). In addition, host defense peptides (HDPs), produced mainly by intestinal epithelial cells and phagocytes in the gastrointestinal tract, are important components of the innate immune system that play critical roles in pathogen elimination. These HDPs can be stimulated by nutritional compounds, including vitamin D_3_, butyrate, and zinc, in addition to infection and inflammation (Talukder et al., 2011; Zeng et al., 2013; Merriman et al., 2015). Recent studies have revealed that probiotics can stimulate HDP expression without modulating inflammatory responses (Schlee et al., 2008; Liu et al., 2017). However, different probiotic strains show varying HDP-inducing abilities, and the HDP secretion induction and antibacterial activity of secreted HDP have not yet been studied.

*Lactobacillus* is among the predominant indigenous genera in human and animal gastrointestinal tracts and is commonly used in probiotics. Our previous studies revealed that dietary supplementation with *L. plantarum* ZLP001, originally isolated from the intestinal tract of a healthy weaned piglet (Wang et al., 2011), exerted beneficial effects on growth performance and antioxidant status in weaning piglets (Wang et al., 2012). However, the potential inhibitory impact on pathogenic bacterial growth and adhesion, and the induction of antimicrobial peptides by this strain are still under investigation. In this study, *L. plantarum* ZLP001 was evaluated for its ETEC growth and adhesion inhibitory abilities as well as for its ability to stimulate the expression and secretion of HDPs and thus enhance the antimicrobial activity of epithelial cell culture supernatant after incubation with *L. plantarum* ZLP001.

## Materials and Methods

### Bacterial Culture

*Lactobacillus plantarum* ZLP001 was isolated from a healthy piglet in our laboratory, identified by the China Center of Industrial Culture Collection (Beijing, China), and preserved in the China General Microbiological Culture Collection Center (CGMCC No. 7370). *L. plantarum* ZLP001 were grown in improved De Man Rogosa Sharpe (MRS) medium at 37°C under anaerobic condition.

The *E. coli* used in our study was an F4-expressing ETEC strain (serotype O149:K91, K88ac) obtained from the China Veterinary Culture Collection Center. F4^+^ ETEC were grown in Luria-Bertani (LB) medium (Oxoid, Basingstoke, United Kingdom) at 37°C.

### Antimicrobial Activity Assay

The pathogen growth inhibition by *L. plantarum* ZLP001 were investigated according to the method of Benavides et al. (2016) with some modifications. After overnight culture, *L. plantarum* ZLP001 was inoculated at 1:100 (v/v) in improved MRS liquid medium and cultured for 18 h at 37°C under anaerobic condition. The supernatant and bacterial cells were collected by centrifugation at 4000 ×*g* for 10 min at 4°C. The supernatant was sterilized using a 0.25-μm filter (Corning Inc., Corning, NY, United States). Bacterial cells of *L. plantarum* ZLP001 were washed with phosphate-buffered saline (PBS) and then resuspended to original concentration. To determine the antimicrobial activity of ZLP001, the indicator ETEC strain was grown using LB broth and adjusted to a concentration of 10^7^ colony-forming units (CFU)/mL with LB broth. This prepared culture was poured on pre-prepared nutrient agar plates containing several Oxford cups, which were removed when the agar was solidified. The *L. plantarum* ZLP001 culture solution, supernatant, and resuspended bacterial cells (100 μL) were spotted onto the wells and incubated at 37°C. After 12-h incubation, the inhibition zones were determined. Three independent experiments were carried out. The mean diameters of inhibition zones were estimated, and inhibition halos >15 mm indicated high inhibitory activity (Benavides et al., 2016).

### Coculture of *L. plantarum* ZLP001 and ETEC

After overnight culture, *L. plantarum* ZLP001 and ETEC were diluted to 10^7^ CFU/mL in sterile MRS medium, which equally supports the growth of *L. plantarum* and ETEC. Then, 10^7^ CFU *L. plantarum* ZLP001 and ETEC were inoculated together in fresh MRS medium to a final volume of 50 mL. One milliliter of pure culture samples and 1 mL of co-culture samples were collected after 6, 12, and 24 h of incubation to evaluate bacterial growth. Samples were spread in dilutions of 10^-1^–10^-6^, and *L. plantarum* ZLP001 and ETEC on MRS and LB agar plates, respectively, and incubated at 37°C for colony enumeration. The pH of the samples was measured at different intervals.

### Cell Line and Culture Conditions

The porcine intestinal epithelial cell line IPEC-J2 was originally derived from jejunums of neonatal piglets (Schierack et al., 2006) and is considered a valuable *in vitro* model system for investigating the interaction of bacteria (commensal or transient) with the small intestinal epithelium. The IPEC-J2 cells used in the present study were purchased from JENNIE-O Biological Technology (Guangzhou, China). IPEC-J2 cells were cultured in a 1:1 mixture of Dulbecco’s modified Eagle’s medium/Ham’s Nutrient Mixture F-12 (DMEM/F12) supplemented with 10% fetal bovine serum (FBS), streptomycin (100 μg/mL), and amphotericin B (0.5 μg/mL) under 5% CO_2_ in a 95% air atmosphere with 90% humidity at 37°C. The cells were maintained by replacing the medium with fresh medium every 2–3 days and were split with 0.25% w/v trypsin (Gibco-Invitrogen, Carlsbad, CA, United States) at each passage.

### Adhesion and Adhesion Inhibition Assays

Adhesion of *L. plantarum* ZLP001 to IPEC-J2 cells was evaluated by microscopy and agar plate counting. IPEC-J2 cells were seeded into 6-well plates at a density of 2.5 × 10^5^ cells/well (Costar, Corning Inc., Corning, NY, United States). When the cells had grown to ∼80% confluence (approximately overnight), they were exposed to *L. plantarum* ZLP001 at different concentrations (10^7^, 10^8^, and 10^9^ CFU/mL). *L. plantarum* ZLP001 bacteria were resuspended and diluted in DMEM/F12 without FBS or antibiotics. The plates were incubated for 2 h at 37°C. All assays were replicated in duplicate wells. Treated IPEC-J2 cells were washed three times with PBS, fixed with methanol, followed by gram staining, and then sealed with resin (Sigma-Aldrich, St. Louis, MO, United States). Adhered *L. plantarum* ZLP001 were observed by microscopy at a magnification of 1,000×. The number of bacteria adhered per 100 IPEC-J2 cells was counted and is reported as the adhesion index. Agar plate counting was performed according to the method described by Kaushik et al. (2009). After incubation, the supernatant was discarded and the cells were washed with PBS. After the cells were lysed with 100 μL of 0.2% Triton^TM^ X-100 (Sigma-Aldrich, St. Louis, MO, United States), viable counts of *L. plantarum* ZLP001 were determined by serial dilution and plating on MRS agar. Adhesion was expressed as bacteria adhering to IPEC-J2 cells per well.

To evaluate the inhibitory effect of *L. plantarum* ZLP001 on ETEC adhesion to IPEC-J2 cells, *L. plantarum* ZLP001 was added at 10^7^, 10^8^, and 10^9^ CFU/mL 1 h before (pre-addition), at the same time (co-addition), or 1 h after (post-addition) the indicator ETEC strain was added. Cells treated with only ETEC were used as a control. After 2 h of incubation, the IPEC-J2 cells were washed to remove unbound bacteria. The cells were lysed with 100 μL of 0.2% Triton^TM^ X-100, and viable ETEC counts were determined by serial dilution and plating on LB agar. Adhesion was calculated as the percentage of adhering ETEC normalized to the control.

### Detection of HDP and Proinflammatory Cytokine Expression by Real-Time PCR

To evaluate the stimulatory effects of *L. plantarum* ZLP001 on HDP and proinflammatory cytokine expression in IPEC-J2 cells, the cells were incubated with or without *L. plantarum* ZLP001 at different concentrations. The concentrations for concentration-dependent experiments were 10^5^, 10^6^, 10^7^, 10^8^, and 10^9^ CFU/mL *L. plantarum* ZLP001. DMEM/F12 containing different concentrations of *L. plantarum* ZLP001 was obtained as described above. The incubation time was set at 6 h after a time-dependent (3, 6, 9, and 12 h) preliminary experiment (data not shown).

After treatment, the cells were lysed directly in RNAzol (Molecular Research Center, Cincinnati, OH, United States) to extract total RNA, according to the manufacturer’s instructions. RNA concentration and purity were determined using a NanoDrop Spectrophotometer (NanoDrop Technologies, Inc., Wilmington, DE, United States). First-strand cDNA was synthesized by reverse transcription of 1 μg of total RNA using an iScript^TM^ cDNA Synthesis Kit (Bio-Rad Laboratories, Inc., Hercules, CA, United States), according to the manufacturer’s instructions. Real-time PCR was carried out on a QuantStudio 3 Real-Time PCR System (Applied Biosystems, Foster City, CA, United States) with iTaq^TM^ Universal SYBR^®^ Green Supermix (Bio-Rad Laboratories, Inc., Hercules, CA, United States). The porcine-specific primers used in this study were designed using the Primer Express software (Applied Biosystems, Foster City, CA, United States). The expression level of each gene was normalized to that of the housekeeping gene glyceraldehyde-3-phosphate dehydrogenase (*GAPDH*). All primers used in this study are listed in **Table 1**. The ΔΔCt method as described by Livak and Schmittgen (2001) was used to calculate relative gene expression.

**Table 1:** Primer sequences used for quantitative real-time PCR.

Gene	Forward primer	Reverse primer	Product size (bp)	Accession number
*GAPDH*	GCTACACTGAGGACCAGGTTG	CCTGTTGCTGTAGCCAAATTC	146	XM_021091114.1
*pBD-1*	TTCCTCCTCATGGTCCTGTT	AGGTGCCGATCTGTTTCATC	130	NM_213838.1
*pBD-2*	TGTCTGCCTCCTCTCTTCC	AACAGGTCCCTTCAATCCTG	149	NM_214442.2
*pBD-3*	CCTTCTCTTTGCCTTGCTCTT	GCCACTCACAGAACAGCTACC	163	XM_021074698.1
*PG1-5*	ACGGTGAAGGAGACTGTG	CGCAGAACCTACGCCTACAA	196	XM_021070622.1
*pEP2C*	ACTGCTTGTTCTCCAGAGCC	TGGCACAGATGACAAAGCCT	92	XM_003362076.4
*IL-6*	AAATGCTCTTCACCTCTC	TCACACTTCTCATACTTCTC	106	NM_001252429.1
*IL-8*	TTCGATGCCAGTGCATAAATA	CTGTACACCTTCTGCACCCA	176	NM_213867.1
*TNF-α*	CCCCTCTGAAAAAGACACCA	TCGAAGTGCAGTAGGCAGAA	180	NM_214022.1


### Enzyme-Linked Immunosorbent Assay (ELISA) of Porcine β-Defensin 1 (pBD-1) and pBD-2

From each treatment described as above, 500 μL of cell culture supernatant was collected, centrifuged at 4,000 ×*g* for 10 min at 4°C, and passed through a 0.25-μm filter. Secreted pBD-1 and pBD-2 were quantified using commercial ELISA kits (Cloud-Clone Corp. USCN Life Science, Inc., Wuhan, China), according to the manufacturer’s protocols.

### Antibacterial Activity of the Cell Culture Supernatant

Antibacterial activity of the cell culture supernatant was determined refer to the method by Wan M.L. et al. (2016). Prepared IPEC-J2 cells were treated with *L. plantarum* ZLP001 at different concentrations (10^5^, 10^6^, 10^7^, 10^8^, and 10^9^ CFU/mL) in triplicate. DMEM-F12 containing different concentrations of *L. plantarum* ZLP001 was prepared as described above. Non-treated IPEC-J2 cells were used as a negative control. Wells containing only *L. plantarum* ZLP001 at different concentrations resuspended in DMEM/F12 were used as positive controls to subtract any influence of *L. plantarum* ZLP001 metabolites on antimicrobial activity. After 6 h of incubation, the cell culture supernatant was collected and passed through a 0.25-μm filter. The indicator ETEC strain was used to evaluate the antibacterial activity of the supernatant. Overnight-grown ETEC was harvested by centrifugation, washed three times in PBS, and resuspended to a final concentration of 10^7^ CFU/mL. Ten microliters of ETEC suspension was incubated with 500 μL of cell culture supernatant. After 2 h of incubation at 37°C with shaking at 200 rpm, the number of viable ETEC bacteria was quantified by serial dilution and plating on LB agar.

### Statistical Analysis

Statistical analysis was performed using one-way analysis of variance (ANOVA) and Student’s *t*-test in the SAS statistical software package version 9.3 (SAS Institute Inc., Cary, NC, United States). Duncan’s multiple range test was performed to compare the differences between means (Harter, 1960). GraphPad Prism version 5 (GraphPad Software, Inc., San Diego, CA, United States) was used to visualize the data. The level of confidence at which experimental results were considered significant was *P* < 0.05.

## Results

### *L. plantarum* ZLP001 Exhibits Antimicrobial Activity

**FIGURE 1:**
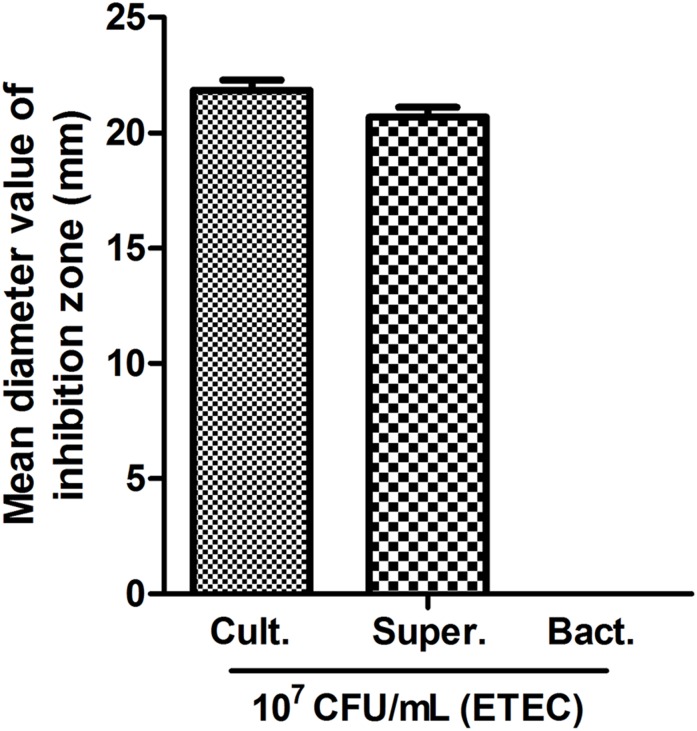
Antimicrobial activity of *L. plantarum* ZLP001. Mean inhibition zone diameter in mm recorded after 12 h of incubation. Bars represent means ± standard errors (SEs) of three independent experiments.

*Lactobacillus plantarum* ZLP001 bacterial culture solution and supernatant exhibited antimicrobial activity against 10^7^ CFU/mL ETEC, with mean inhibition-zone diameters of 21.8 and 20.7 mm, respectively (**Figure 1**). No inhibition zone was observed with *L. plantarum* ZLP001 resuspended in PBS (the inhibition zone diameter was approximately 0–1 mm). **Supplementary Figure S1** visualizes an inhibition zone formed by *L. plantarum* ZLP001 on 10^7^ CFU/mL ETEC.

### *L. plantarum* ZLP001 Inhibits ETEC Growth

**FIGURE 2:**
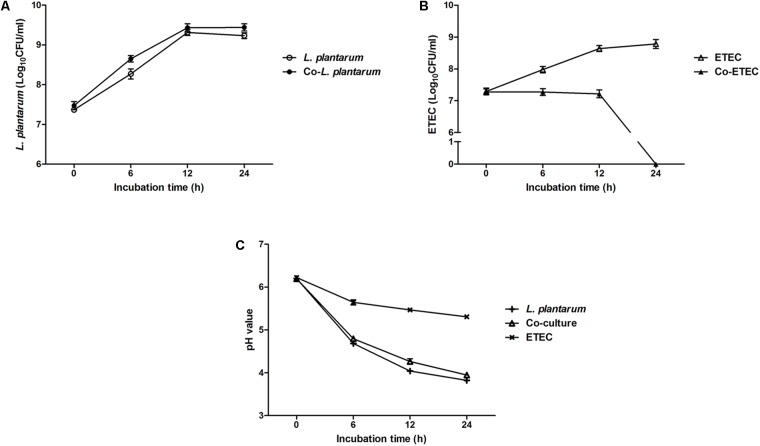
Growth of *L. plantarum* ZLP001 **(A)** and ETEC **(B)** in pure culture and co-culture in MRS medium, and pH of *L. plantarum* ZLP001 and ETEC **(C)** pure culture and co-culture medium. Data are presented as means ± SEs of three independent experiments.

**Figure 2** shows the growth patterns of *L. plantarum* ZLP001 and ETEC in pure culture and in co-culture. The viable count of each species and medium pH were measured. *L. plantarum* ZLP001 showed similar growth patterns in pure culture and co-culture (**Figure 2A**). The number of viable cells was slightly higher in co-culture than in pure culture. ETEC grew in MRS medium and reached a concentration of 10^8^ CFU/mL at the end of culture (24 h) (**Figure 2B**). ETEC growth was inhibited in co-culture with *L. plantarum* ZLP001, and the viable counts were constant until 12 h and then rapidly declined. After 24 h, no viable ETEC bacteria were detected. Acid production by *L. plantarum* ZLP001, as indicated by a decrease in medium pH (**Figure 2C**), was the same under each of the culture conditions. The decline in pH in pure ETEC culture was slower than that in pure *L. plantarum* ZLP001 culture and co-culture, with the pH dropping to 5.32 at the end of the culture (24 h).

### *L. plantarum* ZLP001 Adheres to Porcine Intestinal Cells and Inhibits ETEC Adhesion

**FIGURE 3:**
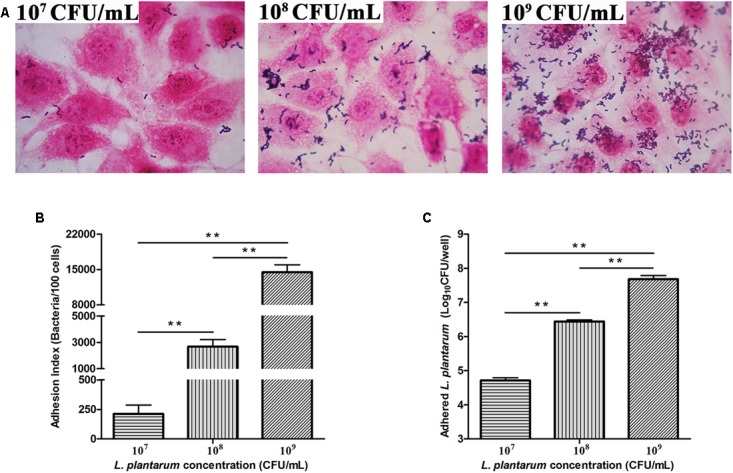
Adhesion of *L. plantarum* ZLP001 to porcine small intestinal epithelial cells (IPEC-J2) **(A)**. Adhesion of *L. plantarum* ZLP001 to IPEC-J2 cells as indicated by adhesion index, which represents the number of adhered *L. plantarum* ZLP001 to 100 cells **(B)** and count of adhered viable *L. plantarum* ZLP001 **(C)**. Cells were incubated with *L. plantarum* ZLP001 at different concentrations (10^7^,10^8^, and 10^9^ CFU/mL) for 2 h. The magnification was 1,000×. Values are presented as means ± SEs of three independent experiments. ^∗∗^*P* < 0.01 compared to each concentration.

Adhesion of *L. plantarum* ZLP001 to IPEC-J2 cells was observed by light microscopy after methanol fixation and Gram staining (**Figure 3A**). The adhesion index showed an obvious concentration-dependent effect (*P* < 0.01); the number of *L. plantarum* ZLP001 cells adhered to 100 IPEC-J2 cells sharply increased with inoculated bacterial concentration (**Figure 3B**). The adhesion capacity as assessed by the agar-plate counting method was also concentration-dependent (*P* < 0.01). The adhered *L. plantarum* ZLP001 increased from 4.72 log CFU at 10^7^ inoculated bacteria to 7.68 log CFU at 10^9^ inoculated bacteria (**Figure 3C**).

**FIGURE 4:**
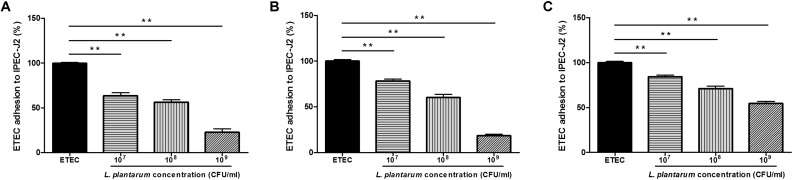
Inhibitory effect of *L plantarum* ZLP001 on ETEC adhesion to IPEC-J2 cells. ETEC adhesion inhibition was determined upon incubation in medium containing ∼10^7^, ∼10^8^, and ∼10^9^ CFU/mL of *L. plantarum* ZLP001 added 1 h before (pre-addition, **A**), at the same time (co-addition, **B**), or 1 h after (post-addition, **C**) addition of ETEC. Values are presented as means ± SEs of three independent experiments. ^∗∗^*P* < 0.01 compared to ETEC treatment.

To investigate the inhibitory effects of *L. plantarum* ZLP001 on ETEC adhesion, a high (10^9^ CFU/mL), intermediate (10^8^ CFU/mL), and low concentration (10^7^ CFU/mL) of *L. plantarum* ZLP001 were tested. *L. plantarum* ZLP001 inhibited ETEC adhesion at all concentrations (**Figure 4**, *P* < 0.01) in a concentration-dependent manner, and the inhibition ratio increased considerably with inoculated bacterial concentration. To compare different inhibition assays, *L. plantarum* ZLP001 was added to IPEC-J2 cells 1 h before (pre-addition, **Figure 4A**), simultaneously with (co-addition, **Figure 4B**), or 1 h after (post-addition, **Figure 4C**) addition of ETEC. The results showed that *L. plantarum* ZLP001 inhibited ETEC adhesion regardless of the time of administration. Post-addition had a lesser inhibitory effect than pre- and co-addition. When data obtained for the three concentrations were pooled, ETEC adhesion was 47.4% for the pre-addition assay, 52.3% for the co-addition assay, and 70.0% for the post-addition assay.

### *L. plantarum* ZLP001 Induces HDP mRNA Expression in Porcine Intestinal Cells

**FIGURE 5:**
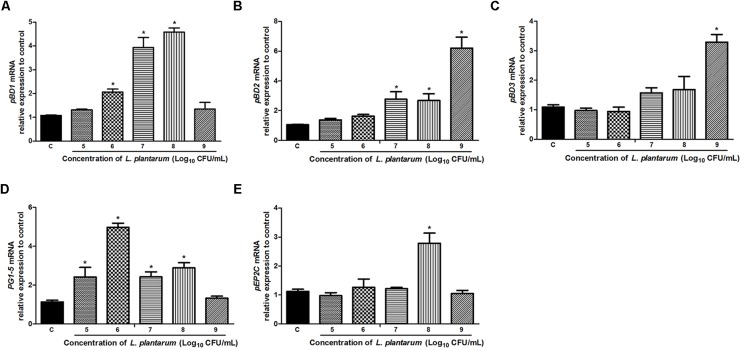
Relative gene expression of *pBD1* **(A)**, *pBD2* **(B)**, *pBD3* **(C)**, *PG1-5* **(D)**, and *pEP2C* **(E)** induced by *L. plantarum* ZLP001 in IPEC-J2 cells. Cells were incubated with *L. plantarum* ZLP001 at different concentrations (10^5^,10^6^,10^7^,10^8^, and 10^9^ CFU/mL) for 6 h. mRNA expression was standardized to *GAPDH* expression. The relative fold changes versus the unstimulated control were calculated by the ΔΔCt method. Values are presented as means ± SEs of three independent experiments. ^∗^*P* < 0.05 compared to non-stimulated control. C, non-stimulated control.

The mRNA expression of porcine HDPs was measured in IPEC-J2 cells to assess the effects of *L. plantarum* ZLP001 on HDP modulation. We detected most of the porcine HDP genes, including the two main families in mammals (cathelicidins and β-defensins), by real-time PCR (**Figure 5**). HDP genes with significantly induced expression in IPEC-J2 cells upon exposure to *L. plantarum* ZLP001 included *pBD1*, *pBD2*, *pBD3*, protegrins 1–5 (*PG1–5*), and epididymis protein 2 splicing variant C (*pEP2C*). Genes showing undetectable expression levels before or after treatment, undetectable expression levels in unstimulated cells, or no significant difference in expression levels after incubation were excluded from further analysis. Most genes with significant induction following exposure of IPEC-J2 cells to *L. plantarum* ZLP001 showed concentration-dependence. The expression levels of *pBD2* (**Figure 5B**) and *pBD3* (**Figure 5C**) obviously increased along with *L. plantarum* ZLP001 concentration, with the highest fold inductions at 10^9^ CFU/mL (*P* < 0.05). For *pBD1* (**Figure 5A**) and *PG1-5* (**Figure 5D**), the expression levels first increased and then tended to decrease, with mRNA expression of *pBD1* peaking at 10^8^ CFU/mL and that of *PG1-5* at 10^6^ CFU/mL. Additionally, *pEP2C* (**Figure 5E**) mRNA expression was maximal at 10^8^ CFU/mL, while other concentrations did not induce an obvious increase (*P* > 0.05). The magnitude of induction also varied among several genes; *pB*D2 showed the highest maximum fold change, whereas *pEP2C* showed the lowest maximum fold change.

### *L. plantarum* ZLP001 Does Not Induce Proinflammatory Cytokine mRNA Expression in Porcine Intestinal Cells

Relative mRNA expression of interleukin 6 (*IL-6*), *IL-8*, and tumor necrosis factor α (*TNFα*) induced by *L. plantarum* ZLP001 in porcine IPEC-J2 cells was determined (**Supplementary Figures S2A–C**). The results showed that none of the detected proinflammatory cytokines were induced by *L. plantarum* ZLP001 inoculation, regardless of concentration (*P* > 0.05), which suggested that *L. plantarum* ZLP001 did not provoke an inflammatory response in the intestine.

### *L. plantarum* ZLP001 Induces HDP Secretion by Porcine Intestinal Cells

**FIGURE 6:**
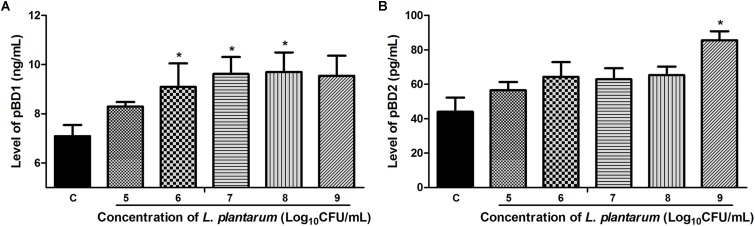
Levels of pBD1 **(A)** and pBD2 **(B)** protein in IPEC-J2 cell-culture supernatant following treatment of the cells with *L. plantarum* ZLP001 (10^5^, 10^6^, 10^7^, 10^8^, and 10^9^ CFU/mL) for 6 h. Protein levels in the supernatant were determined by ELISA. Data are presented as means ± SEs of three independent experiments. ^∗^*P* < 0.05 compared to non-stimulated control. C, non-stimulated control.

The antibacterial effect of *L. plantarum* ZLP001 on IPEC-J2 cells against ETEC may be associated with the HDP expression and secretion. We investigated the levels of pBD1 and pBD2 (commercial ELISA kits with antibodies for other HDPs were not available) in the cell-culture supernatant using ELISA after treatment of cells with *L. plantarum* ZLP001 at different concentrations (**Figure 6**). *L. plantarum* ZLP001 showed different abilities to promote pBD1 and pBD2 secretions compared to that of the control at different concentrations. For pBD1 (**Figure 6A**), high concentrations of inoculated *L. plantarum* ZLP001 induced significantly increased defensin secretion (10^7^–10^9^ CFU/mL, *P* < 0.05), whereas for pBD2 (**Figure 6B**), only the highest inoculated concentration showed a significant induction of secretion (*P* < 0.05).

### Antibacterial Activity of Cell-Culture Supernatant

**FIGURE 7:**
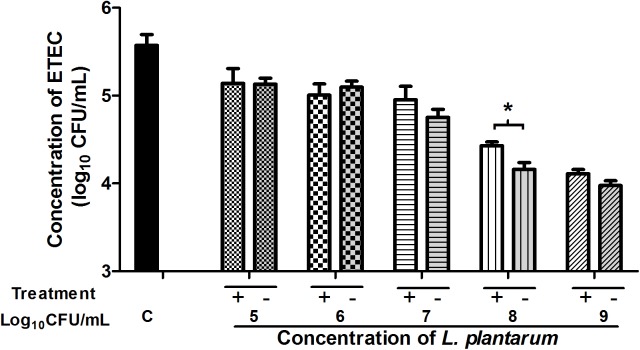
Antibacterial activity of IPEC-J2 cell-culture supernatants collected after incubation with *L. plantarum* ZLP001 at different concentrations (10^5^, 10^6^, 10^7^, 10^8^, and 10^9^ CFU/mL) for 6 h or supernatants of *L. plantarum* alone incubated in DMEM)/F12. Values are expressed as the number of viable enterotoxigenic ETEC present after 2 h incubation in the supernatant in three independent experiments. C, non-stimulated cell control (negative control); Treatment +, supernatant of *L. plantarum* ZLP001 culture in DMEM/F12 (positive control); Treatment –, cell-culture supernatant after *L. plantarum* ZLP001 incubation. ^∗^*P* < 0.05.

To further evaluate the antibacterial effects of *L. plantarum* ZLP001 after stimulation of IPEC-J2 cells, the antibacterial activity of cell-culture supernatant was measured using the indicator ETEC strain (**Figure 7**). IPEC-J2 cells were incubated in the absence or presence of *L. plantarum* ZLP001 at different concentrations. Considering the proliferation and antibacterial activity of *L. plantarum* ZLP001 itself, we inoculated *L. plantarum* ZLP001 alone in DMEM/F12 as a positive control. Supernatant collected from *L. plantarum* ZLP001-treated IPEC-J2 cells reduced ETEC counts compared to the negative control (without *L. plantarum* ZLP001) at all concentrations of ZLP001, and further reduced the counts as compared to supernatant collected from *L. plantarum* ZLP001 alone, at the concentration of 10^8^ CFU/mL(*P* < 0.05).

## Discussion

Many *L. plantarum* have been studied extensively and shown to possess broad-spectrum antimicrobial properties in different hosts (Guerra-Ordaz et al., 2014; El Halfawy et al., 2017). However, the mechanisms underlying pathogen inhibition and interaction with the host are still not thoroughly understood. In order to explain the mode of action of this species, antimicrobial properties were evaluated from different perspectives.

Growth inhibition of harmful bacteria is a major property of probiotics. The present study indicated that *L. plantarum* ZLP001 inhibited the growth of the common intestinal pathogen ETEC based on inhibition zone and co-culture assays. *Lactobacillus* spp. have the ability to upregulate host antimicrobial factors (Kirjavainen et al., 2008), which is possibly related to the lactic acid they produce, low pH, and antimicrobial compounds (Longdet et al., 2011; Benavides et al., 2016). Acidic environment and stress induction in the outer membrane are all factors that potentially affect ETEC survival (Delley et al., 2015). In the present study, the *L. plantarum* ZLP001 bacterial culture as well as the supernatant showed antagonistic activity against ETEC, while no antagonistic activity (no inhibition zone) was observed with bacterial cells. This result suggested that the antimicrobial activity of *L. plantarum* ZLP001 is mainly related to its metabolism or the low pH condition rather than the bacteria *per se*. In our co-culture assay, the decreasing trend of pH under co-culture of *L. plantarum* ZLP001 and ETEC was similar to that observed for *L. plantarum* ZLP001 cultured alone. However, the negative effects of *L. plantarum* ZLP001 on viable ETEC count were not as pronounced when the co-culture period was less than 12 h. This suggested that the inhibitory effects of *L. plantarum* ZLP001 on ETEC viability occurred mainly via antibacterial metabolism. This observation is supported the finding that acidic conditions mediated by lactic acid are not the predominant mechanism by which *Lactobacilli* probiotics act (Fayol-Messaoudi et al., 2005). *Lactobacillus* can produce broad-spectrum antimicrobial substances, such as extracellular organic acids, hydrogen peroxide, and bacteriocin-like compounds, which act against gram-positive and gram-negative pathogens (Azizi et al., 2017; Wang et al., 2017). We previously assessed the production of lactic acid by *L. plantarum* ZLP001 after 24 h of fermentation, which was in the range of 50–60 mmol/L (data not published). In addition, we demonstrated that *L. plantarum* ZLP001 has the ability to produce hydrogen peroxide based on DAB staining intensity. With respect to bacteriocin, the presence of structural genes encoding for plantacirin in this strain as well as the antimicrobial agents secreted by *L. plantarum* ZLP001 remain to be confirmed.

Adhesion property is considered one of the most essential factors for a probiotic to fulfill its beneficial function. This study demonstrated that *L. plantarum* ZLP001 can effectively adhere to IPEC-J2 cells, which is consistent with the findings of a previous study using other *Lactobacillus* strains on IPEC-1 cells (Wang et al., 2016). The concentration of inoculated *L. plantarum* ZLP001 and the number of adhered viable bacteria or the adhesion index showed a clear concentration-dependent relationship. However, pathogen adhesion is a prerequisite for the initiation of the infection, which is associated with the destruction of the intestinal epithelial structure and is crucial for targeted delivery of secreted enterotoxins. Besides producing enterotoxin, F4-fimbriated ETEC specifically attach to receptors on the brush border of the mucosa by expressing F4 fimbrial adhesins, which initiate infection (González-Ortiz et al., 2013). Several studies have demonstrated that probiotics have the potential to prevent infection by inhibiting pathogen adhesion and penetration (Zareie et al., 2006; Fukuda et al., 2011). Our present results suggested that *L. plantarum* ZLP001 has the ability to inhibit ETEC adhesion to IPEC-J2 cells. This inhibition was more effective when *L. plantarum* ZLP001 was added at higher concentrations. Similar results have been reported by Jin et al. (2000), who showed that *Enterococcus faecium* 18C23 effectively inhibited the adhesion of *E. coli* F4ac to piglet intestinal immobilized mucus, especially at 10^9^ CFU or more. Probiotics can pre-occupy or compete for pathogen-binding sites, thus interfering with pathogen adhesion and colonization (Tuomola et al., 1999). Our results were similar to those from other reports demonstrating that inhibition by probiotic added to epithelial cells prior to pathogens is more effective than attempting to disrupt established pathogen colonization (Dunne et al., 2014; Manning et al., 2016). This suggested that *L. plantarum* ZLP001 can prevent ETEC adherence mechanistically through steric hindrance or binding-site competition (Wong et al., 2013). The binding sites are composed of different types of molecules, like fibronectin and collagen. *Lactobacillus* species have the ability to bind these molecules (Lorca et al., 2002; de Leeuw et al., 2006). In addition, bacterial co-aggregation may inhibit adhesion. Further, secretion of bacteriocin and other antimicrobial substances may be involved (Lebeer et al., 2010).

Host defense peptides exert both antimicrobial and immunomodulatory activities, and contribute to epithelial innate immune defense (Bevins et al., 1999; Zhang et al., 2000). The antimicrobial activity of HDPs is associated with the intestine microbiota and protects the host against pathogens, including bacteria, fungi, and viruses (Smet and Contreras, 2005; Veldhuizen et al., 2008). Enhancing the synthesis of endogenous HDPs is beneficial to the early response to infection and inflammation (Veldhuizen et al., 2008). Probiotic microbes are able to induce HDP production, including in pigs (Borchers et al., 2009; Wan L.Y. et al., 2016; Liu et al., 2017). We observed that *pBD1* mRNA expression was significantly upregulated after exposure to *L. plantarum* ZLP001, which is inconsistent with the results of Liu et al. (2017), who showed that *pBD1* mRNA expression was not significantly increased in IPEC-J2 cells or piglets exposed to *Lactobacillus reuteri* I5007. The potency of probiotics to modulate HDP production varies among strains (Schlee et al., 2008), which may result in different efficacies of different strains. Upregulation of *pBD2* in response to *L. plantarum* ZLP001 likely inhibited pathogenic bacteria in our study, as reported previously that pBD2 protects against a wide range of pathogenic bacteria *in vitro* (Veldhuizen et al., 2007; Zhang et al., 2011; Deng et al., 2013). pBD3 exhibits not only profound antimicrobial properties, but also strong immunoregulatory ability by regulating the expression of the proinflammatory cytokine IL-8 (Dou et al., 2017). In the present study, *pBD3* mRNA expression was considerably increased only at the highest concentration of *L. plantarum* ZLP001, which was inconsistent with another report of high *pBD3* expression at 10^7^ and 10^8^ CFU/mL (Liu et al., 2017). Furthermore, increases in the expression of other antimicrobial peptides (*PG1-5* and *pEP2C*) were observed in the present study, accounting for the *L. plantarum* ZLP001-mediated protective effects against pathogen infection. In addition to defense-response modification, HDPs are also correlated with nutrient digestibility, intestinal morphology, and growth performance in weaning pigs (Yoon et al., 2013). This implies that *L. plantarum* ZLP001-induced HDP gene expression may be beneficial not only to the innate immune response, but also to body health and production performance.

Our results suggested that *L. plantarum* ZLP001 enhances the intestinal defense response via induction of HDP secretion. To our knowledge, this is the first study to illustrate that *Lactobacillus* can stimulate porcine HDP secretion in intestinal epithelial cells. Only one previous study using human intestinal epithelial cells (Caco-2) showed induction of defensin secretion by the probiotic *Lactobacillus fermentum* and *E. coli* (strain Nissle 1917) (Schlee et al., 2008). Similarly, this is the first study to show ETEC-antimicrobial activity of cell-culture supernatant post-*Lactobacillus* treatment. The results obtained in our study were not as pronounced as those reported by Wan M.L. et al. (2016) who used cell-culture supernatant from epigallocatechin-3-gallate (EGCG)-treated cells and observed 32% lower *E. coli* counts than those in the control. EGCG has no antibacterial effects on *E. coli*. In contrast, the stimulator strain used in our study possesses strong antibacterial activity *per se*. Thus, maybe the effective positive control to evaluate the antimicrobial effect of the cell culture supernatant after *L. plantarum* ZLP001 treatment was insufficient. However, based on our results, it can be concluded that the antibacterial activity of *L. plantarum* ZLP001-stimulated IPEC-J2 cell-culture supernatant was not due to *L. plantarum* ZLP001 *per se*, but rather was a result of antimicrobial substances being secreted into the supernatant by IPEC-J2 cells induced by *L. plantarum* ZLP001. Certainly, further studies are required to verify the antimicrobial activities of the HDPs secreted by probiotic-induced epithelial cells. The effectiveness of probiotics in innate immune defense is an important starting point for future deeper studies of the benefits of probiotics to intestinal health and against infection.

## Conclusion

In conclusion, we demonstrated that *L. plantarum* ZLP001 possesses antimicrobial activity. It can prevent ETEC growth by producing certain antimicrobial substances in combination with generating a relatively acidic environment. *L. plantarum* ZLP001 adhered to IPEC-J2 cells and inhibited ETEC adhesion mainly through exclusion and competition. *L. plantarum* ZLP001 also induced the expression and secretion of HDPs in intestinal epithelial cells, thus enhancing the antimicrobial activity of cell-culture supernatant after *L. plantarum* ZLP001 incubation. These functions of *L. plantarum* ZLP001 may account for its protective effects against pathogenic infection. Thus, *L. plantarum* ZLP001 may prove useful as a probiotic strain in piglet production. However, the lack of *in vivo* experiments was a limitation of the present study and thus, further studies *in vivo* are essential to verify the protective effect of *L. plantarum* ZLP001 and to delineate the exact underlying mechanism.

## Author Contributions

JW and HJ conceived and designed the study. JW was responsible for the bacterial and cell assays, data analysis, and writing. HJ conceived and designed the experiments. YZ participated in the adhesion inhibition assay. SW participated in the ETEC growth inhibition assay. HL participated in the real-time PCR test. WZ participated in the bacterial adhesion assay. DZ and YW participated in the ELISA.

## Conflict of Interest Statement

The authors declare that the research was conducted in the absence of any commercial or financial relationships that could be construed as a potential conflict of interest.

## Supplementary Material

FIGURE S1 Visualization of the inhibition zone produced by *L. plantarum* ZLP001 toward enterotoxigenic *Escherichia coli* (ETEC). Cult., culture solution; Super., supernatant; Bact., bacteria; CFU, colony- forming unit.

FIGURE S2 Relative gene expression of interleukin 6 (*IL-6*, **A**), *IL-8* **(B)**, and tumor necrosis factor α (*TNFα*, **C**) induced by *Lactobacillus plantarum* ZLP001 in porcine small intestinal epithelial cells (IPEC-J2). Cells were incubated with *L. plantarum* ZLP001 at different concentrations (10^5^,10^6^,10^7^,10^8^, and 10^9^ CFU/mL) for 6 h. mRNA expression was standardized to glyceraldehyde-3-phosphate dehydrogenase (*GAPDH*) expression. The relative fold changes versus the unstimulated control were calculated with the ΔΔCt method. Values are presented as means ± standard errors of three independent experiments. C, unstimulated control; CFU, colony-forming unit.
