# Supplementary figures and images for: Swine-Derived Probiotic Lactobacillus plantarum Inhibits Growth and Adhesion of Enterotoxigenic Escherichia coli and Mediates Host Defense
Source: Front Microbiol. 2018 Jun 26;9:1364. doi: 10.3389/fmicb.2018.01364 (PMC6028558; doi:10.3389/fmicb.2018.01364)

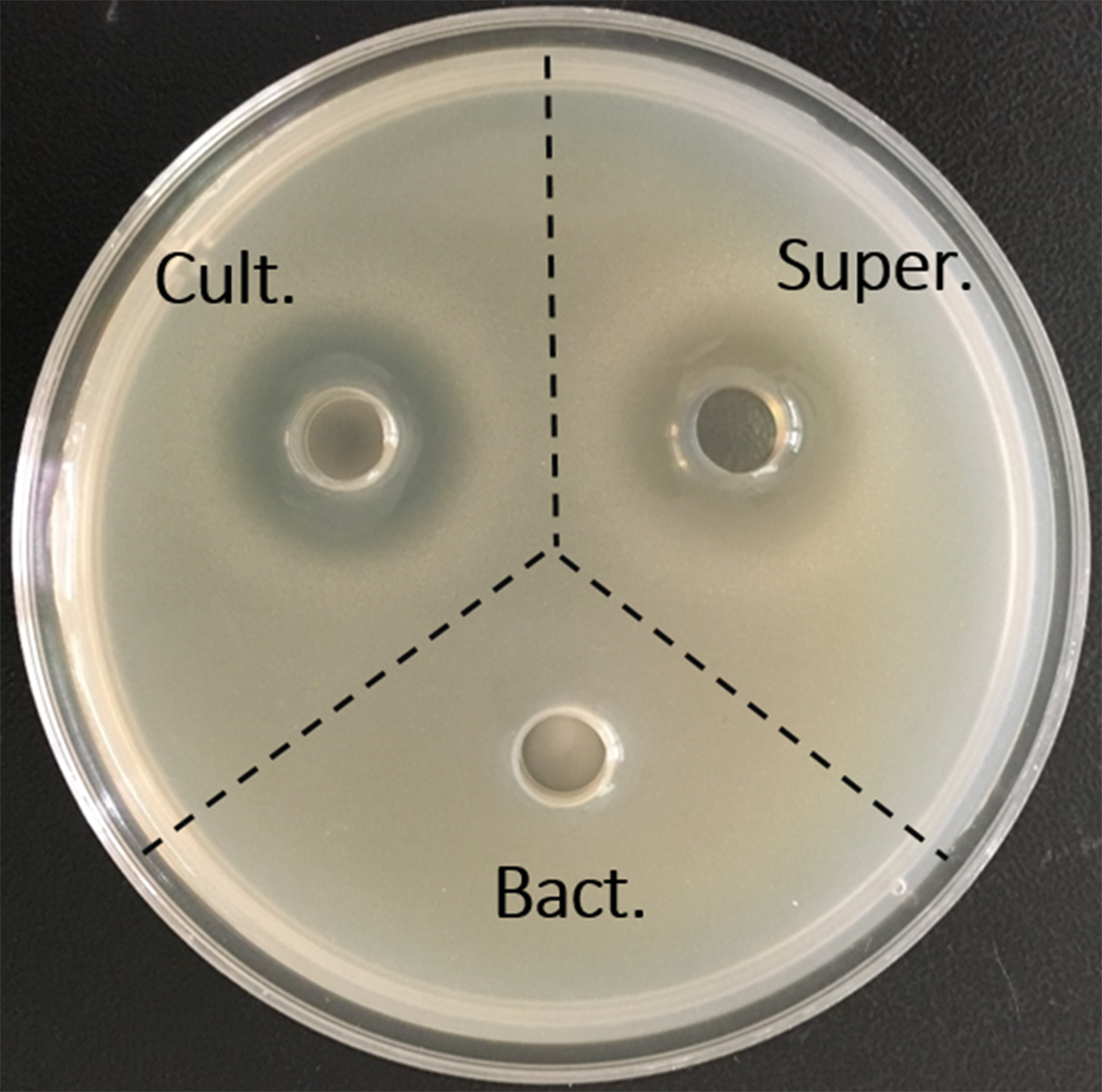

Supplement: FIGURE S1 — Visualization of the inhibition zone produced by L. plantarum ZLP001 toward enterotoxigenic Escherichia coli (ETEC). Cult., culture solution; Super., supernatant; Bact., bacteria; CFU, colony- forming unit. [file Image_1.tif]

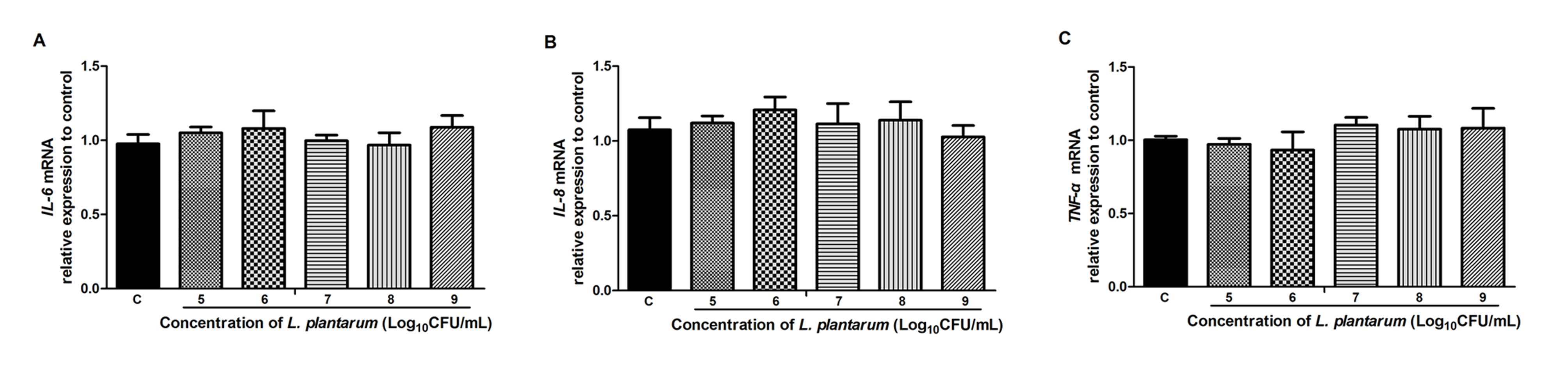

Supplement: FIGURE S2 — Relative gene expression of interleukin 6 (IL-6, A), IL-8 (B), and tumor necrosis factor α (TNFα, C) induced by Lactobacillus plantarum ZLP001 in porcine small intestinal epithelial cells (IPEC-J2). Cells were incubated with L. plantarum ZLP001 at different concentrations (105,106,107,108, and 109 CFU/mL) for 6 h. mRNA expression was standardized to glyceraldehyde-3-phosphate dehydrogenase (GAPDH) expression. The relative fold changes versus the unstimulated control were calculated with the ΔΔCt method. Values are presented as means ± standard errors of three independent experiments. C, unstimulated control; CFU, colony-forming unit. [file Image_2.tif]
